# What Have Data Standards Ever Done for Us?

**DOI:** 10.1016/j.mcpro.2025.100933

**Published:** 2025-02-28

**Authors:** S.E. Orchard

**Affiliations:** European Molecular Biology Laboratory, European Bioinformatics Institute (EMBL-EBI), Wellcome Genome Campus, Hinxton, Cambs, UK

**Keywords:** data standards, Human Proteome Organization, Proteomics Standards Initiative, molecular interactions, protein–protein interactions, protein complexes

## Abstract

The Human Proteome Organization Proteomics Standards Initiative has been successfully developing guidelines, data formats, and controlled vocabularies for both the field of molecular interaction and that of mass spectrometry for more than 20 years. This review explores some of the ways that the proteomics community has benefitted from the development of community standards and takes a look at some of the tools and resources that have been improved or developed as a result of the work of the Human Proteome Organization-Proteomics Standards Initiative.

## Highlights

• The Human Proteome Organization Proteomics Standards Initiative publishes data standards for experimental protein science.
• Standards allow loss-free data transfer between instruments, software, and databases.
• Data repository Consortia merge diverse datasets using standard data formats.
• Data generators need to improve dataset annotation to support successful reanalysis.

## Graphical Abstract

**Figure:**
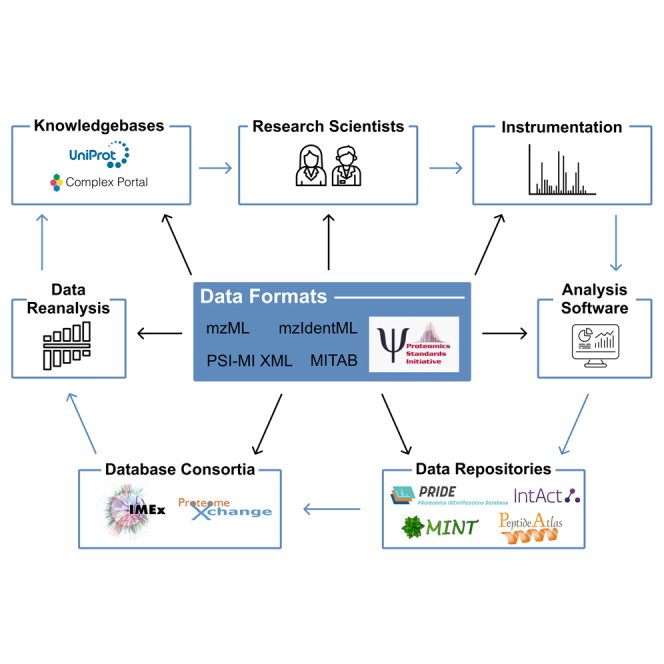

## In Brief

The HUPO-Proteomics Standards Initiative (PSI) publishes guidelines, data formats, and controlled vocabularies describing molecular interactions and mass spectrometry experiments. These standards have enabled the transfer of information between data generation, analysis, and repositories. Users have benefitted from an ever-increasing volume of consistently formatted and annotated information available for reanalysis. One route this has been made available to users is via knowledge bases such as UniProt which imports information on protein interactions, posttranslation modifications, and protein translation evidence from large-scale data Consortia.

Many of the more significant advances in biomedical science made in the last few decades, and those that we anticipate being made in this era of machine-learning and artificial intelligence, can be directly attributed to the vast quantities of data which are now freely available to researchers in their laboratory or on their home computer. These data have been contributed by other workers in the same field who have deposited their results in open-source repositories, making it available for use in consistent data formats and have annotated it to uniform community-driven standards. However, this has not always been the case. In the early days of large-scale data generation, efforts to concatenate the results in common repositories were hampered by the fact that the information was often only made available in researcher-specific or vendor-dictated formats. There was also suspicion that releasing raw data could result in its misinterpretation by a third party or lead scientists to act as “research parasites” reinterpreting published data to fit their own agenda, to disprove the original author’s theory, or just pre-empting the original author from further publication on the original dataset (1). During this period, data was often siloed in local databases and only a subset of information shared via a pdf file published in the Supplementary Materials attached to a publication. Moving data into any analysis tool generally requires some degree of data transformation, usually inevitably accompanied by information loss.

Clearly this had to change. The example set by repositories such as wwPDB (2) and the International Nucleotide Sequence Database Consortium (3), both of whom have made data freely available for more than 40 years showed the biomedical/biological community examples of good data sharing practice and the benefits it could bring to the community as a whole. The existence of such resources enabled the building of KnowledgeBases possible, for example UniProtKB (4), which takes information from these repositories, for example protein sequences, and augments these with information extracted from the literature and from other databases. Both International Nucleotide Sequence Database Consortium and wwPDB made their information available in a single common file format so that it could be readily merged and integrated into other resources.

The next significant step forward in the availability of open source, constantly formatted data was taken in 2001 in the field of microarrays when *Brazma et al.* published a set of guidelines for describing “the minimum information required to ensure that microarray data can be easily interpreted and that results derived from its analysis can be independently verified.” (5) This led to the development of formats for capturing the output of experiments involving microarrays and the establishment of public domain data repositories for its storage, paving the way for many other fields to follow.

In the early days of mass spectrometry–based proteomics, the vendor-dictated format ruled. This often locked the users of a specific type of mass spectrometer into a limited choice of search engine to perform their peptide/protein identifications, as each search engine could only read a limited number of input file types. No data repositories existed, and the concept of data reanalysis or data reuse was unheard of. The field of molecular interactions, specifically that of protein–protein interactions, was in a somewhat different position in that several repositories were established in the early 2000s, including BIND (6), DIP (7), and MINT (8), but each of these had developed their own independent data format. There was also no agreement on protein identifiers, with BIND using NCBI gi numbers, DIP mainly ReSeq identifiers, and MINT UniProt accession numbers. All of this made data integration across resources near impossible. The development of network visualization and analysis tools was also severely compromised as each tool developed worked only on a single data source. Some databases were looking to move beyond the simple “A interacts with B” level of data capture, but there was little point in annotating, for example, the details of a binding domain into a database if the information could not be subsequently visualized or mapped onto a protein interaction network. It was becoming increasingly obvious that proteomics researchers needed to agree on formats and standards which were freely available to the community and could be adopted by researchers, instrument manufacturers, software producers, and repositories alike.

## The Human Proteome Organization

The Human Proteome Organization (HUPO) Proteomics Standards Initiative (HUPO-PSI) has been responsible for creating, maintaining, and promoting data standards in the field of protein science since 2002 (9). This group was tasked by HUPO with ensuring that proteomics data could be made freely available, downloadable, and transferable between different data resources and tools. Over the last 20+ years, the initiative has organized an annual Spring Workshop at which new developments in the relevant fields are identified, discussed, and the need for any new or updated standards agreed upon. The HUPO-PSI is organized as a set of working groups and an overall steering group. The domain-specific working groups are responsible for the design and implementation of data standards relevant to that area. The need for broad community buy-in to this process was recognized right from the very beginning of this collaborative effort and various strategies have been put in place over the years to ensure this happens. These have included presentation of the concept at relevant conferences, direct invitation to experts in the field to be part of the process, feedback mechanisms organized in collaboration with relevant journals, and a formal document process developed by the HUPO-PSI (10). Input from industry has been actively sought and both instrumentation manufacturers and software vendors have contributed over the years. These measures ensure that the specifications, informational and community practice documents are exposed to as many members of the community as possible prior to final publication.

The work of the HUPO-PSI has been well documented over the years (9, 10). The aim of this review is to visit a few exemplar cases where proteomics standards have been developed, implemented, and have influenced downstream work and resources, in particular in my own area of interest, that of molecular interactions.

## The Molecular Interaction Working Group

The Molecular Interaction (MI) Working Group was initially focused on standardizing the ever-increasing amount of protein–protein interaction data being generated. Over the years, however, its remit has broadened and now the formats and standards are flexible enough to describe interaction between any molecular type, up to and including macromolecular complexes. As with all HUPO-PSI work groups, its output falls into three categories

(i) A minimum requirements document which describes the information required for a reader of a publication to understand and potentially reproduce an experiment and for a successful deposition to a database to be made. The Minimum Information about a Molecular Interaction experiment document published in 2007 has been regularly reviewed but remains pertinent to data reported, to date (9, 11).
(ii) Data formats enabling the transfer of information between resources and tools. The MI group currently supports two XML formats. PSI-MI XML2.5 (12) enables the capture of experimental data linked to a single publication, including full details of the experiment and molecule constructs such as protein tags, binding regions, and the effects of point mutations to the amino acid sequence of one or more of the participants. The more flexible PSI-MI XML3.0 (13) enables the description of more abstracted data derived from multiple publications. Examples of the latter can include an interaction which has an allosteric effect on an enzyme or the representation of the components, stoichiometry, and topology of a protein complex. It can also deal with more complex experimental data such as the output of a kinetics experiment where the strength of an interaction, or the list of involved participants, varies according to different point mutations. A simpler tab-limited format (MITAB) allows the transfer of a subset of these data but is adequate to the needs of most researchers looking to perform network analysis on large-scale datasets (14).
(iii) Controlled vocabularies/ontologies to annotate the data captured in databases and in the data formats. The PSI-MI controlled vocabulary (www.ebi.ac.uk/ols4/ontologies/mi) contains the terms required to describe all aspects of a molecular interaction experiment and to enable the management of a database containing such data. It is continually updated, currently containing more than 1450 terms, and the addition of new terms has enabled the data formats to remain relatively constant. For example, the development of proximity biotinylation (15) in 2014 resulted in new interaction detection method terms (proximity-dependent biotin identification; MI:1314) and interaction type (proximity; MI:2364) rather than any changes to the data formats.

## The Mass Spectrometry Proteomics Working Group(s)

The MS proteomics group was also a founding work group of the HUPO-PSI in 2002. At around the same time, the domain-specific journal “*Molecular & Cellular Proteomics*” also recognized this urgent need to set standards on how to present proteomic data, launching the first guidelines in 2004 (16).

The number of HUPO-PSI MS Proteomics working groups has varied as the discipline has matured and new techniques have replaced earlier ones, with journal guidelines evolving in parallel (17, 18). The documents and specifications released by these contributors include the following:

(i) The Minimum Information About a Proteomics Experiment (MIAPE) guidelines which were developed in a modular structure, such that each aspect of a proteomics experiment would correspond to a module (19). Modules have included the description of the mass spectrometry (MIAPE-MS) (20), the downstream informatics analysis (MIAPE-MSI) (21), and any quantitative components (MIAPE-Quant) (21, 22). In practice, the information requested on these documents to accompany any publication or database deposition has largely overlapped with the publication requirements set out by individual journals, so compliance to the technical requirements of these standards in the published literature is generally good. However, many submitters are still failing to fully, or even partially, provide “meta-data” that is,. to annotate experimental detail such as sample characteristics and how these relate to the data in the deposition files. The lack of such key information limits the reusability of these datasets by researchers and is a major hurdle this community still needs to address.
(ii) The primary standardized format for encoding the output of mass spectrometer instruments is mzML originally published in 2009 but since updated (23). mzIdentML enables the encoding of peptide/protein identifications from an MS proteomics experiment, and this has been widely adopted by software tools and data repositories. A simpler tab-delimited version (mzTAB) of this format has also been developed and is widely used for database depositions. New formats under development include a JSON-based format for encoding mass spectrometry quality control metrics (mzQC) (24) and a spectral library format (mzSpecLib) which focuses on the consistent capture of associated metadata. The Universal Spectrum Identifier has been created by this group as a multipart key that can be used in publications to identify and retrieve specific spectra and peptide-spectrum matches in databases (24, 25).
(iii) The PSI-Mass Spectrometry controlled vocabulary (www.ebi.ac.uk/ols4/ontologies/ms) contains terms that describe all aspects of an MS-based proteomics experiment (26). It encompasses terms for a complete MS analysis pipeline, including sample labeling, digestion enzymes, instrumentation, software for peptide/protein identification and quantification, and parameters for significance determination. Again, support for newer technologies, such as data independent acquisition and ion mobility spectrometry, was largely achieved by extending PSI-Mass Spectrometry rather than by changing the format.

## What has the Research Community Gained from Implementing Data Standards

The work of the HUPO-PSI has been described many times, but the tangible benefits of these efforts are less well documented. The data standards have been adopted by many different proteomics and molecular interaction databases and data resources. A general acceptance that the benefits of data deposition far outweigh any perceived issues has led to a huge increase in the amount of both MS and molecular interaction data freely available in the public domain. Consortia have been established as databases share the load of storing and annotating this information. The availability of consistently formatted, well annotated data has, in turn, resulted in a proliferation of tools developed for its visualization and analysis with the days of one tool per dataset now long since a thing of the past. The analyses performed by these tools are routinely captured by other data resources and KnowledgeBases which make the information derived from these datasets more widely available and display them in the context of other related datasets.

## The IMEx Consortium–Shared Infrastructure and Shared Curation Effort

The IMEx Consortium of Molecular Interaction databases was formed to share curation practices and exchange interaction datasets. It now makes a single, unified dataset available via both the IMEx website (www.imexconsortium.org) and that of many of its participating members (IntAct, MINT, DIP) (27). Other contributing members display only a subset of data, for example, UniProtKB imports a scored and filtered high-quality set, with no restriction on species, displayed as Binary Interactions in relevant records whereas MatrixDB (28) only takes data relating to the extracellular matrix proteins, proteoglycans, and polysaccharides from a limited number of species. The data is imported by many other resources, such as the Human Protein Atlas (29), STRING (30), Reactome (31), and the Gene Ontology (32). Since its inception, the Consortium has moved from simple cohosting of interaction records curated by different curation teams to sharing of tools and infrastructure. The IMEx members play different roles and share the tasks required for running the IMEx consortium. The IntAct database provides a web-based editorial platform for collaborative curation by all IMEx partners and also provides long-term data storage and maintenance. MINT database develops and maintains the PSI-MI controlled vocabularies, while the DIP database handles the curation tracker site IMEx Central. The importance of this collaborative effort has been globally recognized and has resulted in the IMEx Consortium being awarded both Global Core Biodata Resource (https://globalbiodata.org/what-we-do/global-core-biodata-resources/) and Elixir Core Data Resource (https://elixir-europe.org/platforms/data/core-data-resources) status.

The IMEx curation model is very detailed, with both full experimental detail (experimental organism, interaction detection, and participant identification methodologies) and participant details (native vs expressed, construct details such as tags, binding region, site-directed mutations) being captured. The description of biologically relevant information enables the construction of more sophisticated networks with, for example, the effect of a specific disease-causing mutation of a local protein network be analyzed and visualized (33). The data is imported into UniProtKB where it is visualized using the ProtVista Feature Viewer and can be aligned with information about that specific amino acid location from other sources. For example, meta-data such as cell/tissue type, involvement in disease, and the details of agonists can also be described within the data formats and are leading to the possibility of building context-specific networks. For example, the interactome of a specific cell type or a comparison of a physiological versus pathological disease network.

### ProteomeXchange

The MS proteomics standards have led to a revolution in data deposition with the ProteomeXchange (PX) consortium of proteomics resources created in 2012 to enable submission and dissemination of public MS proteomics data (34). Members include the PRIDE database (EMBL-EBI) (35), the PASSEL resource (35) within PeptideAtlas (36) (Institute for Systems Biology), MassIVE (University of California), jPOST (37) (Japan), iProX (38) (National Center for Protein Sciences), and Panorama Public (39) (University of Washington). PeptideAtlas also participates by reanalyzing public submitted datasets. As of September 2024, around 55,000 datasets had been submitted to PX resources and this number continues to grow. Submission to a PX database is now an accepted part of the publication process for the MS proteomics community. The increasing volume of open-source data availability has led to a concomitant increase in the download, reanalysis, and reuse of this information. Data has been used to improve gene models in genomic databases, to verify protein sequences, to look for expression patterns across different tissue types within an organism, and also increasingly in machine-learning and artificial intelligence algorithms.

## Tool Development

Most of the tools built by active members of the HUPO-PSI are aimed at enabling data deposition, enhancing data findability, and encouraging data reuse and reanalysis. This is not intended to be an exhaustive catalog of these resources, rather a summary of some of the more significant developments.

## Molecular Interaction Tools and Resources

The first significant tool developed as a result of the MI HUPO-PSI standards and formats was the Proteomics Standard Initiative Common QUery InterfaCe (PSICQUIC) (40). This enables computational access to molecular-interaction data resources by means of a standard Web Service and query language. PSICQUIC defines a minimum set of standard SOAP (Simple Object Access Protocol) and REST (REpresentational State Transfer) methods to be implemented by every molecular-interaction provider. These methods accept a MIQL (Molecular Interactions Query Language) query as input and return MI information in one of the standard formats as output. PSICQUIC is currently used as the foundation on which several websites are built, examples include that of the IMEx Consortium and the MINT database. It also enables users of Cytoscape (41) to query back from existing networks to expand selected nodes using additional data from any resource supporting a PSICQUIC service.

**Fig. 1:**
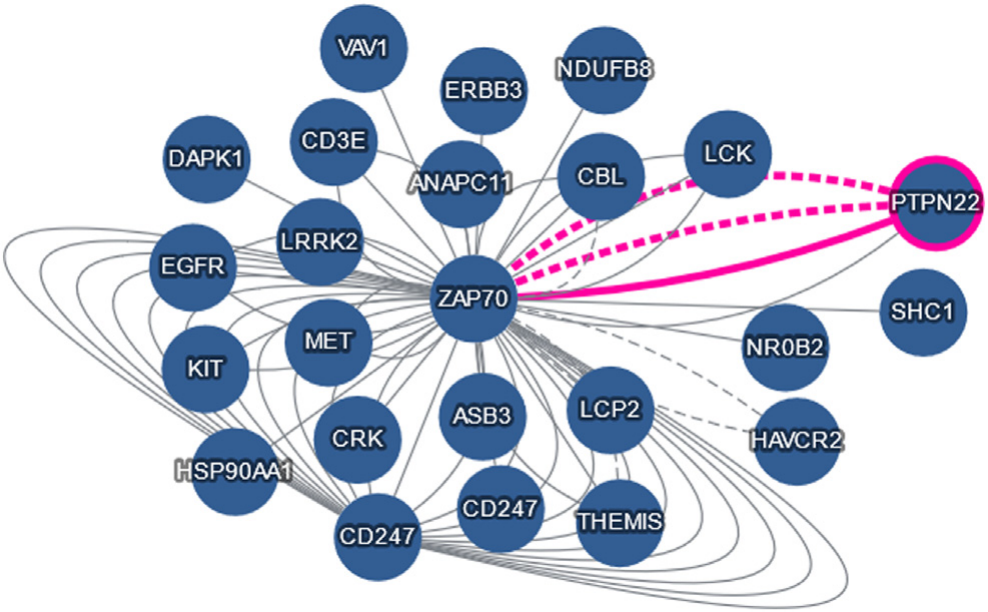
**IntAct view of the human proteins known to interact with ZAP70 (**P43403**).** The *pink* lines indicate an interaction affected by a site-directed mutation.

However, as noted above, IMEx data contains a wealth of additional information including details of binding regions, required posttranslational modifications, and the effect of both site-directed and deletion mutations (Fig. 1). More sophisticated access to the IMEx dataset is available via the Cytoscape App developed by the IntAct Molecular Interaction team at the European Bioinformatics Institute (EMBL-EBI) (42). This provides users with full access to the different layers of IMEx data, ensuring their readability by offering different predefined viewing styles of any network, nested navigation, and filtering capabilities on multiple levels. The IntAct App also supports the creation of sub networks, Cytoscape session saves and its core features are available via command line, thus allowing automation and scripting access (https://ebi-intact.github.io/IntActApp/automation_support).

The EMBL-EBI team also developed the Complex Portal (http://www.ebi.ac.uk/complexportal), a reference resource of manually curated macromolecular complexes that captures the biologically functional units of proteins and other participating molecules, such as cofactors or noncoding RNAs (43). The descriptions of these assemblies include details of stoichiometry, topology, and specific binding regions when available and required a visualization tool that could accurately represent these attributes. Complexes are available for download in PSI-MI XML2.5, the recommended XML3.0, or a tab-delimited format developed specifically for protein complex data, ComplexTAB (44). However, although the PSI-MI XML format fully captures the detail of the interactions, it is not designed for parsing within browsers. To provide the required level of detail in a concise, easy-to-parse format, the JavaScript Object Notation format MI-JSON was created and used in the development of the ComplexViewer (45), a freely available visualization tool which provides a 2D visualization of the topology of each complex. The viewer provides a scalable vector graphic element within a web page designed to display the full detail of a complex or small network and enables zooming into a protein sequence, to see details of a binding region and also the delineation of subcomplexes within a larger assembly. The use of the ComplexViewer has now been adopted by multiple other resources such as UniProtKB and several of the InterMine family of databases all of which import and display complexes from the Complex Portal. It has also been added to the IntAct database website to display molecule features such as binding domains, posttranslational modification, and site-specific amino acid mutations and their effect on a binary interaction or on a small network of interacting molecules.

The MS proteomics standards were designed to support existing instrumentation by providing an open-source alternative to existing formats which would enable more options when writing software and ease interoperability by enabling the chaining of multiple tools to form a seamless workflow for MS spectra processing. Vendor-specific formats are still the option of choice of the mass spectrometer producers but the development of converters has enabled data transfer to search engines and analysis software. Members of the PX Consortium have focused on developing such tools to enable the building of open-source workflows for systematic dataset reanalysis. For example, the formats were incorporated into ProteoWizard (46), an open source set of libraries and tools which aim to support proteomics data manipulation and analysis. PeptideAtlas has released species-specific builds based on PX data for an increasing number of organisms. To enable this, they have developed the Trans-Proteomics Pipeline (47), a set of free and open-source software tools for MS data representation, MS data visualization, peptide identification and validation, protein identification, quantification, and annotation, data storage and mining, and biological inference. Converters within the pipeline enable the conversion of vendor-specific formats and RAW files to HUPO-PSI mzML format. The FragPipe proteomics pipeline and MSFragger accept mzML files as input (48). However, there remain other software tools, MaxQuant (49) and data independent acquisition-NN (50), that do not directly input mzML or return mzIdentML formats, suggesting much work needs to be done to encourage more widespread adoption of these formats. It remains the goal of the HUPO-PSI to work with the vendors and other interested groups to develop a next generation of data exchange formats which will be more broadly adopted and lessen the need for data conversion.

## UniProtKB – a Unifying Resource Benefitting from Data Standardization

**Fig. 2:**
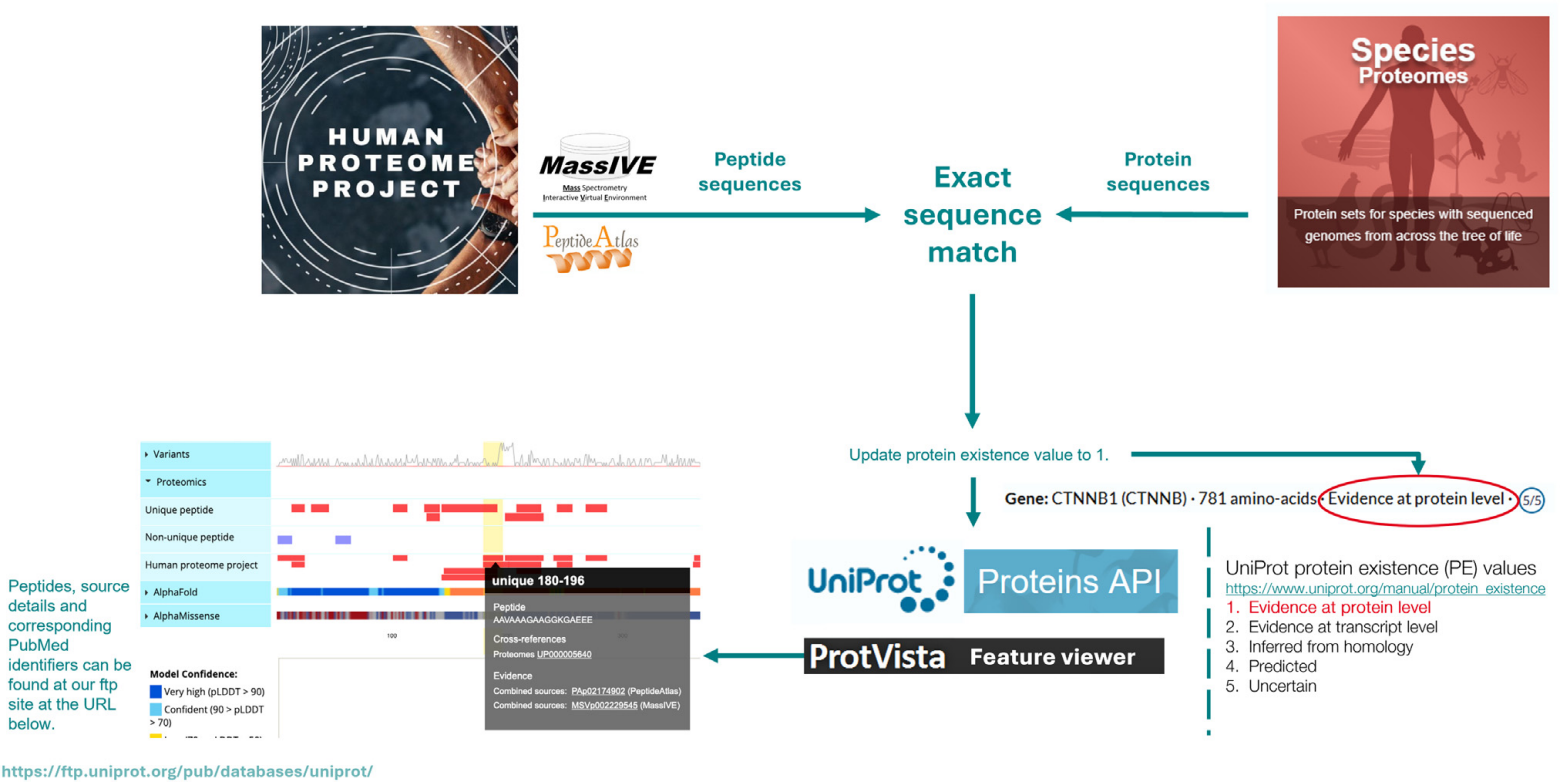
**The HPP peptide pipeline used to confirm the expression of human proteins**.
(ii) Large-scale PTM data is now being added to UniProtKB entries via a pipeline built in collaboration with PRIDE, PeptideAtlas, and the University of Liverpool (53, 54). PTM-specific public proteomics datasets from selected model organisms are filtered, re-analyzed, and site-based confidence scoring applied ensuring that only high-quality datasets are included. These are stored in PRIDE attributed a unique ID (PXD), enabling user access, traceability, and reusability. Modified sites are assigned a confidence score based on their false localization rate across multiple datasets to reflect the strength of evidence available. Data are integrated into UniProtKB and visualized in the ProtVista Feature Viewer embedded in the PTM/Processing section of the protein entry page and also the feature viewer in both site-centric and peptide-centric formats. A Gold/Silver/Bronze notation is awarded to each PTM derived from this pipeline based on its FLR across multiple datasets. Currently, the database contains large-scale protein phosphorylation data for rice (*Oryza sativa* subsp. *japonica*) and *Plasmodium falciparum* with future plans to include data for human, *Arabidopsis thaliana*, yeast (*Saccharomyces cerevisiae*), and mouse. Additional types of PTM will also be added to the pipeline as appropriate datasets become available. The importance of high-quality metadata to enable the selection of suitable datasets cannot be stressed highly enough and the PX have adopted the Sample and Data Relationship Format (SDRF-Proteomics), a tab-delimited text format that describes the relationship between samples and data files (https://github.com/bigbio/proteomics-metadata-standard). Every submission in ProteomeXchange must provide the instrument and result files in addition to the metadata related to the project.
(iii) High-quality binary protein interactions are added to UniProt records from the IMEx Consortium via a pipeline built by the IntAct database. The availability of consistently annotated data in a standard format has enabled the development of MISCORE (55), a customizable, heuristic scoring system that relies on the available annotation evidences associated with an interaction. UniProtKB and IntAct collaborated on developing a version of this to select protein pairs for import into UniProt records and display on the website (for full details, visit https://www.ebi.ac.uk/intact/documentation/user-guide#data_export). Links from the UniProtKB record directs the user back to the original data on the IntAct website.
(iv) As described above, the Complex Portal acts as a reference resource of functional macromolecular assemblies made available in HUPO-PSI MI formats. UniProtKB adds cross-references to the Complex Portal to relevant entries, then uses these to enable both visualization of the complexes using the ComplexViewer described above. Additionally, protein entries which are members of the same complex are grouped in a Search field thus increasing interoperability between entries.

Many resources, some already mentioned, have benefitted from data generated as a direct result of the work of the HUPO-PSI. A detailed look at one specific example allows us to see the scope of these activities. UniProt is the world’s leading high-quality, comprehensive, and freely accessible resource of protein sequence and functional information. The database integrates information on protein sequence, structure, and function from many resources into a single access point for biological scientists. UniProtKB acts both as an information source for proteomics scientists, giving detail of protein sequence, posttranslational modification sites, structure and function, and also imports data from MI and MS proteomics data repositories. Information from pipelines built to reprocess spectra and meta-data from MS repositories or to score and filter data from molecular interaction databases is added to relevant entries to increase our knowledge of specific proteins (51). Standards-compliant data added to UniProt entries include the following:

(i) Peptides from MS studies which are used to confirm the expression of proteins. UniProtKB has recently updated its pipeline to use the HPP3.0 guidelines developed by the HUPO Human Proteome Project, that is, that claims should be supported “by two or more distinct uniquely-mapping, non-nested peptide sequences of length ≥9 amino acids with the above evidence in the same paper”. When 2 peptides overlap, the total extent must be ≥ 18 amino acids” (48). For human entries, UniProtKB now maps peptides from PeptideAtlas and MassIVE to protein sequences which meet these (Fig. 2). The keyword ‘Proteomics identification’ [KW-1267] is now added to both reviewed and unreviewed entries to enable their search and filtering. This work would not be possible without the data deposited into the PX repositories using HUPO-PSI standards and the UniProtKB database now actively supports the work of the HPP which aims to achieve confident detections via mass spectrometry of all human proteins predicted to exist in the human proteome (52).

## Summary

The use of data standards encompassing common formats annotated using ontologies/controlled vocabularies to an agreed specification is now widely accepted in the biomedical research community and has led to a revolution in open data availability. This, in turn, has resulted in an era of transparency and collaboration in data acquisition and data-sharing and is currently engendering a new wave of scientific discovery through the application of machine-learning and artificial intelligence to open-access, high-quality data. For example, the huMap3.0 complexes described above have been predicted by machine-learning from large scale AP-MS and BioID mass spectrometry datasets made available through the PX resources in HUPO PSI data formats (56, 57). The manually curated human complexes from the Complex Portal, which are available in PSI-MI formats, were used as the training/test set. The components, topology, and stoichiometry of these manually curated complexes had been verified, where possible, using data captured by the IMEx Consortium members in standards-compliant datasets. Manually curated *S. cerevisiae* complexes from the same resource had previously been used to evaluate affinity enrichment coupled to mass spectrometry pulldowns of an endogenous GFP-tagged library covering the entire expressed yeast proteome (55), with the resulting main mass spectrometry raw data and MaxQuant output tables deposited to the ProteomeXchange Consortium. Neither of these cross-disciplinary pipelines would have been possible without the development and implementation of data standards.

New data types, methodologies, and instrumentation mean that standards need to be constantly reviewed, updated, or eventually replaced when there is a strong use case and a community interested in working, ideally as part of the HUPO-PSI, to push these forward. The adoption of PSI standards has clear benefits in making proteomics/interactomics data more FAIR (findability, accessibility, interoperability, and reusability) and the development of open-source tools, and data deposition in public domain databases makes all of this globally freely accessible. The HUPO-PSI will therefore continue its efforts to maintain and enhance existing standards to meet emerging needs and call upon the community to adopt these data formats and also to improve the annotation of their own individual experimental output, improving the meta-data to enable and enhance data reuse and reanalysis. We will continue to collaborate with other similar initiatives such that MS proteomics and interactomes data can be readily merged with other data types and further inform our understanding of biology. New contributors are always welcome and invited to attend the Spring workshops and to join relevant Working Groups (https://www.psidev.info/).

## Conflicts of interests

The authors declare that they have no conflicts of interests with the contents of this article.
